# Considerations on effort, precision and accuracy for long‐term monitoring of African lions (*Panthera leo*), when using Bayesian spatial explicit capture–recapture models, in fenced protected areas

**DOI:** 10.1002/ece3.10291

**Published:** 2023-07-17

**Authors:** Isabella A. Ball, David G. Marneweck, Nicholas B. Elliot, Arjun M. Gopalaswamy, Herve Fritz, Jan A. Venter

**Affiliations:** ^1^ Department of Conservation Management, Faculty of Science Nelson Mandela University George South Africa; ^2^ Kameelhoek Farm Kimberley South Africa; ^3^ Conservation Alpha Cape Town South Africa; ^4^ Wildlife Counts Nairobi Kenya; ^5^ Carnassials Global Bengaluru India; ^6^ REHABS International Research Laboratory CNRS‐Université Lyon 1‐Nelson Mandela University George South Africa

**Keywords:** Bayesian spatial explicit capture–recapture, fenced protected area, home range, population density, sampling effort, sex ratio

## Abstract

Intensive management is frequently required in fenced wildlife areas to reduce deleterious effects of isolation. Decisions on how best to manage such wildlife are ideally informed by regular and reliable estimates of spatiotemporal fluctuations in population size and structure. However, even in small, fenced areas, it is difficult and costly to regularly monitor key species using advanced methods. This is particularly the case for large carnivores, which typically occur at low density and are elusive yet are central to management decision‐making due to their top–down effects in ecosystems and attracting tourism. In this study, we aimed to provide robust estimates of population parameters for African lions (*Panthera leo*) and use the data to inform a resource‐efficient long‐term monitoring programme. To achieve this, we used unstructured spatial sampling to collect data on lions in Pilanesberg National Park, a small (~550 km^2^) fenced protected area in South Africa. We used Bayesian spatial capture–recapture models to estimate density, abundance, sex ratio and home range size of lions over the age of 1 year. Finally, to provide guidance on resource requirements for regular monitoring, we rarefied our empirical data set incrementally and analysed the subsets. Lion density was estimated to be 8.8 per 100 km^2^ (posterior SD = 0.6), which was lower than anticipated by park management. Sex ratio was estimated close to parity (0.9♀:1♂), consistent with emerging evidence in fenced lion populations, yet discordant with unfenced populations, which are usually ~2*♀*:1♂ in healthy, source populations. Our rarefied data suggest that a minimum of 4000 km search effort needs to be invested in future monitoring to obtain accurate and precise estimates, while assuming similar detection rates. This study demonstrates an important utility of Bayesian spatial explicit capture–recapture methods for obtaining robust estimates of lion densities and other important parameters in fence‐protected areas to inform decision‐making.

## Graphical abstract

In this study, we aimed to provide robust estimates of population parameters for African lions (*Panthera leo*) and use the data to inform a resource‐efficient long‐term monitoring programme. To achieve this, we used unstructured spatial sampling to collect data on lions in Pilanesberg National Park, South Africa, a small (~550 km^2^) fenced protected area. We used Bayesian spatial capture–recapture models to estimate density, abundance, sex ratio and seasonal home range size of lions over the age of 1 year. Finally, to provide guidance on resource requirements for regular monitoring, we rarefied our empirical data set incrementally and analysed the subsets. Photo credit: Rob Davis.

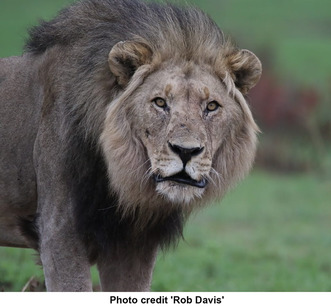

## 1 INTRODUCTION

**FIGURE 1:**
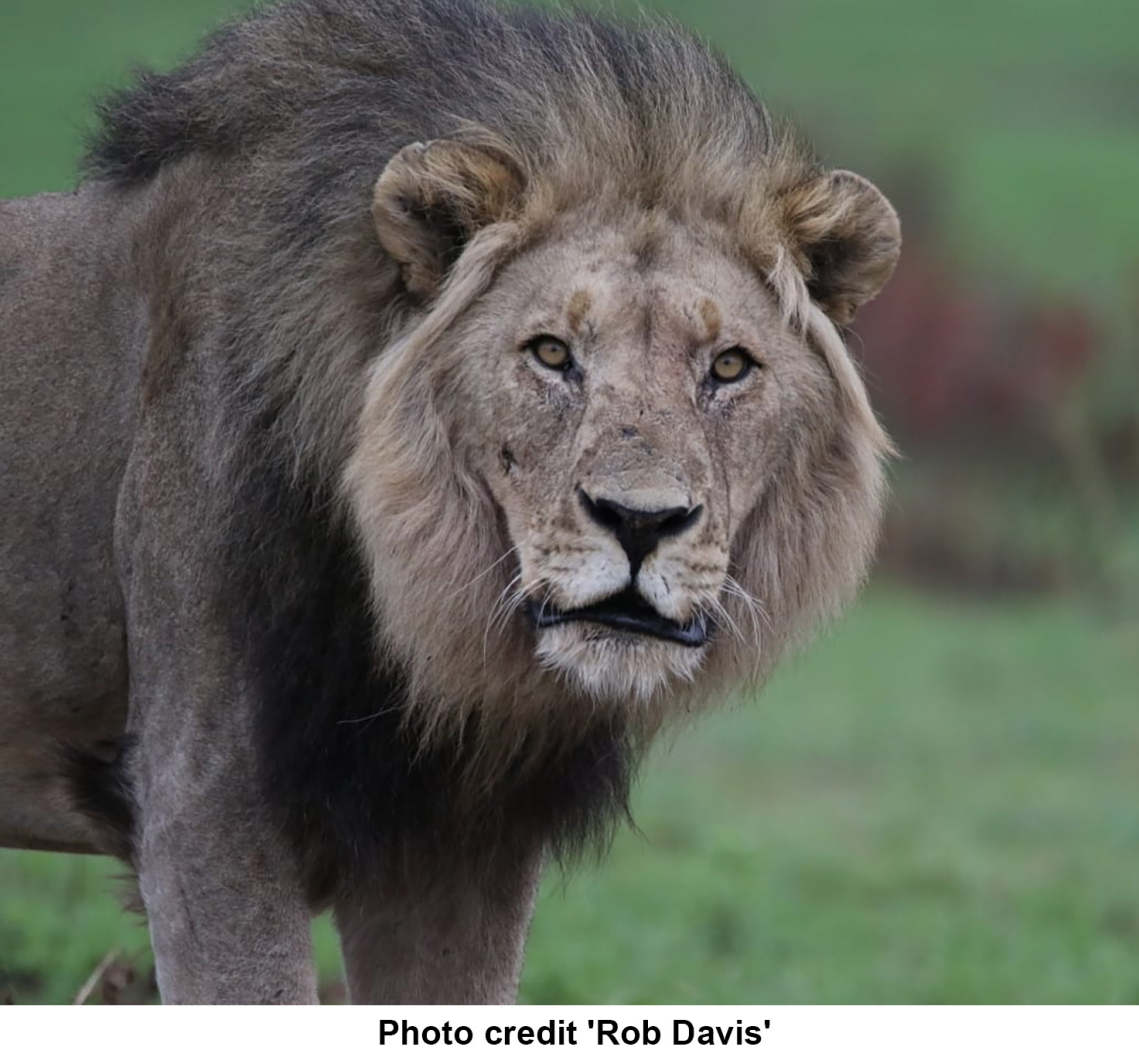
Male African lion (*Panthera leo*) in Pilanesberg National Park, South Africa. Photo credit: Rob Davis.

Large African carnivores, such as lions (*Panthera leo*) (Figure 1), are thought to be declining primarily due to retaliations arising from human–wildlife conflict, and bushmeat poaching resulting in prey depletion (Bauer et al., 2020). These effects appear to be greater in unfenced areas, with one study suggesting that up to half of unfenced lion populations may decline to near extinction (Packer, Loveridge, et al., 2013). Consequently, fencing of wildlife areas in Africa is an increasingly popular, albeit contentious, strategy to ameliorate threats (Creel et al., 2013; Packer, Swanson, et al., 2013; Pekor et al., 2019). While fencing may help to reduce the threats facing free ranging populations, the total isolation of often small populations necessitates intensive management, since confinement and limited space can inhibit dispersal, affect territorial behaviour, cause genetic isolation, lower disease resistance and frequently result in overpopulation (Miller et al., 2013; Miller & Funston, 2014). Lions have direct and indirect top–down regulating effects on prey populations (Kissui & Packer, 2004; Le Roux et al., 2019), which can be exaggerated in small, fenced protected areas as the ability of prey to spatially avoid predators is inhibited (Tambling & Du Toit, 2005). This can cause a ‘predator pit’ (see Clark et al., 2021; Smout et al., 2010) where high predator numbers impact prey species to such an extent that prey populations begin to decline (Clark et al., 2021; Tambling & Du Toit, 2005). This results in significant financial and ecological consequences, which can be reduced by effective management. In South Africa, there are many fenced wildlife areas, and the lions therein are managed as a metapopulation through translocations, contraception or euthanasia, with careful consideration of social structure, population genetics and wildlife numbers (Miller et al., 2013).

Foundational to decision‐making on lion management in fenced areas is the need for accurate and precise estimates of key population parameters, such as abundance and space use. However, estimating these parameters is frequently problematic due to logistical and methodological limitations, even in small, fenced wildlife areas (Braczkowski, Gopalaswamy, Elliot, et al., 2020; Elliot et al., 2020). Traditional methods used to estimate lion population sizes (e.g. call‐up surveys and spoor counts) are often problematic due to confounding effects caused by detection probability, and result in high scientific uncertainty (Gopalaswamy et al., 2015, 2022). Some of these methods are also commonly used in South Africa's protected areas (Ferreira & Funston, 2010; McEvoy, 2019; Tambling & Du Toit, 2005). More recently, Elliot and Gopalaswamy (2017) demonstrated how to apply unstructured spatial sampling to collect individual encounter histories of lions, and fit the data to spatial capture–recapture models. This search encounter‐based SCR (SECR) approach has since been successfully used within many east African source populations to provide robust population estimates and management recommendations (Braczkowski, Gopalaswamy, Nsubuga, et al., 2020; Elliot et al., 2021; Ngene et al., 2023; Western et al., 2022), yet has not been applied in southern Africa.

In our study, we had three objectives: (1) to demonstrate how to apply the SECR approach in a South African setting, where lion populations are intensively managed, yet typically monitored using ad‐hoc methods; (2) to provide the management authority of Pilanesberg National Park, a small, fenced protected area with rigorous estimates of density, abundance, sex ratio and home range size; and (3) to use our empirical data set to inform the creation of a resource‐efficient long‐term monitoring programme that facilitates management decision‐making.

## 2 METHODS

### 2.1 Study area

Pilanesberg National Park (PNP) is located in the Northwest Province of South Africa (−25.2523, 27.0812) and is approximately 550 km^2^. The park is fully enclosed by a predator‐proof electric fence, which effectively confines wildlife within the park (Vanak et al., 2010). After being declared a protected area in 1979 approximately 6000 animals of various taxa were reintroduced, including lions in 1993 (Van Dyk & Slotow, 2003). The animals are well‐habituated to vehicles since PNP is popular among international and local tourists (Stoffelen et al., 2020). Pilanesberg National Park is within the Savanna biome and consists mainly of Pilanesberg Mountain Bushveld veld type (Mucina & Rutherford, 2006) with an average of ~630 mm of rain per year, which falls between September and February (Carruthers, 2011). There are several natural springs and artificial dams that provide perennial water throughout PNP.

### 2.2 Field methods

**FIGURE 2:**
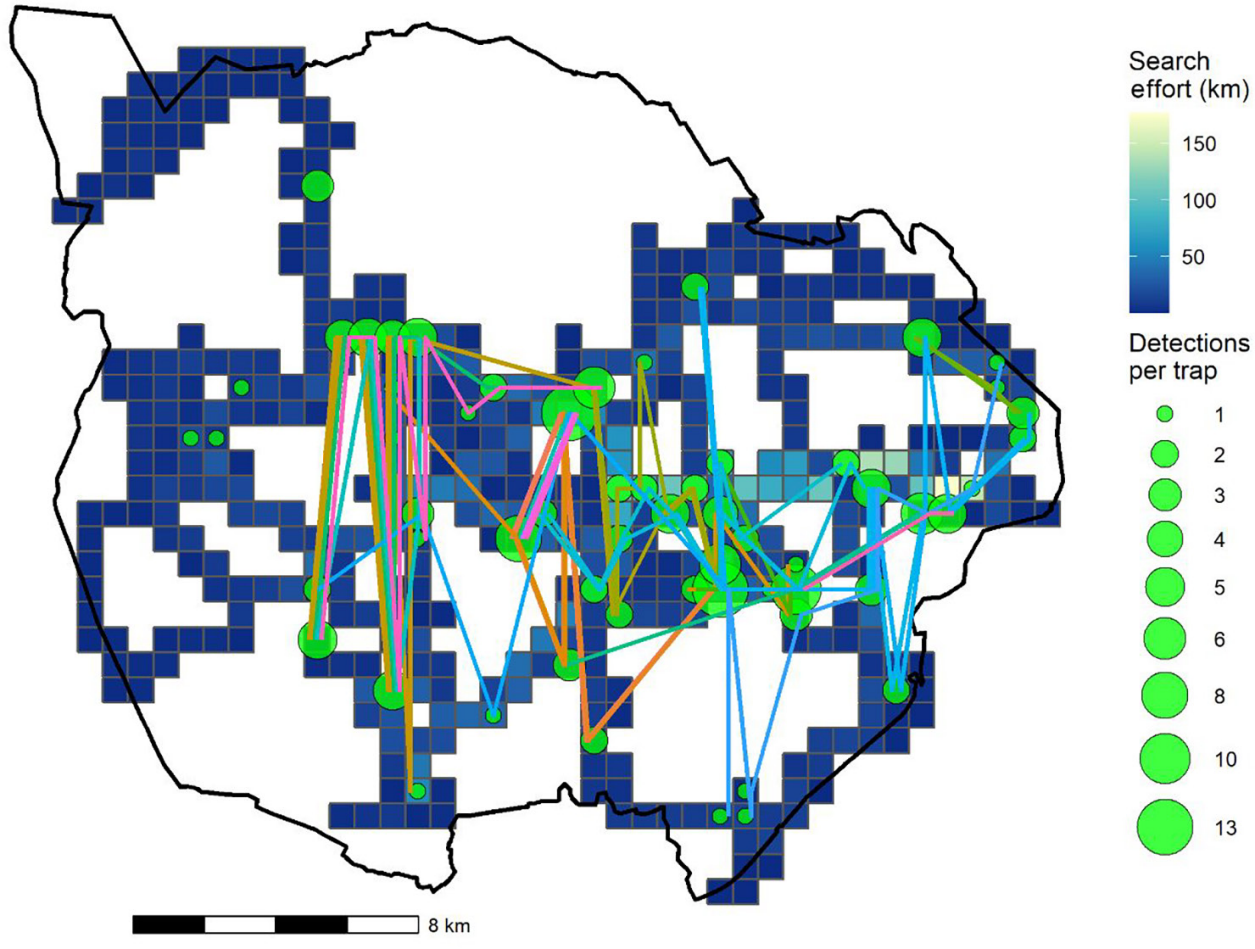
Unstructured spatial sampling protocol was used to find and identify individual lions in Pilanesberg National Park, South Africa. Our spatial capture recapture sampling design accounted for search effort per 0.5 km^2^ pixel (trap) per sampling occasion (1 day) and resulted in 184 detections of 37 individuals (the jittered coloured lines connecting detections represent spatial recaptures of individuals).

All fieldwork was conducted between 25 August 2020 and 10 December 2020. This 108‐day time frame was deemed long enough to obtain a large data set, but short enough to presumably meet assumptions of geographic and demographic closure (Karanth & Nichols, 1998). We used a vehicle to search for lions along tourism and management roads. We searched for lions during the early mornings (05:00–10:30) and late afternoons (16:00–18:30) when lions were most active (Elliot, Cushman, Loveridge, et al., 2014). We used a customised Cybertracker application (www.cybertracker.org), installed on an android smartphone, to record lion locations and search effort, by automatically taking a GPS reading every 10 s. We roughly divided PNP into five road sections, and set routes were driven each morning and afternoon to ensure that our search effort was not biased towards certain areas (Figure 2). Between driving the set routes for the day, we attempted to enhance our lion detections by following up on lion sighting reports (e.g. social media and safari guides). The vegetation in PNP range from open to dense in some areas, which could have influenced detection. Prey species are also not evenly distributed due to vegetation dynamics and burning regimes. The effect of these factors was, however, not tested in this study.

Photographs were taken using a Canon 5D DSLR camera with a Sigma 150–600 mm F/5‐63 lens. Where possible, for each individual sighted, we attempted to take close‐up photographs of the whisker spots on the left and right side of the face, in addition to any unique features (Pennycuick & Rudnai, 1970). We used these photographs to create identity profiles for each individual. Photographs taken at subsequent sightings were then compared with the identity profiles to visually assess whether or not they were the same individual, based on whether or not both sets of whisker spots matched (see Elliot et al., 2020 for more details). Cubs were recorded but lions estimated <1 year were excluded from the analysis due to their potential high mortality rate, which would likely violate the assumption of population closure (Otis et al., 1978). Lions were aged using ageing criteria from Miller and Funston (2016). An external validation of the capture history was performed by one of the authors not involved in the fieldwork. Discrepancies were discussed, and a detection was retained only if both observers agreed on the identity.

### 2.3 Analytical framework

To model the spatial distribution of lions (state process), we generated a state space, which was defined by the park boundary, since PNP is fully enclosed by a predator‐proof electrified fence, which prohibits movement of wildlife to areas outside the park (Vanak et al., 2010). Within the state space, we generated potential activity centres, represented by 0.5 km^2^ pixels. We set the data augmented value of abundance (*M*) to 200, which is the sum of the number of individuals detected during the study (*n* = 37) and the number of individuals augmented for the analysis (*n*_z_ = 163; Royle et al., 2009). Given the fenced nature of PNP, the estimate of abundance (*N*) in the study area is equivalent to (*N*_super_) estimated within the state space. To describe the observation process (the way individual animals were detected), we followed the procedure described by Elliot and Gopalaswamy (2017). This entailed the compilation of a standard SCR matrix, consisting of individuals, sampling occasions and trap locations (pixels of size 0.5 km^2^). Since highly sampled traps might increase the chance of detections, we included an effort covariate (logarithm of kilometres driven per trap, per day). Sex‐specific covariates were included since males and females have different home range sizes, which might affect the observation process. The inclusion of these covariates also allowed us to estimate sex ratio. To provide a measure of sex‐specific home range size, we took the spatial scale parameter (σ) for each sex and applied the formula $\pi {\left(\sigma \sqrt{5.99}\right)}^{2}$ (see page 157 of Royle et al., 2013).

### 2.4 Candidate models

We defined five a‐priori models and compared their posterior outputs (Table 1). We set the detection function parameter (θ) to 1, which implies a fixed, half‐normal detection function. The probability of detecting lion *i* within pixel *j* on sampling occasion *k* is defined by a complementary log–log function of covariates (Elliot & Gopalaswamy, 2017):

$$\text{cloglog}\left(\pi_{\mathrm{ijk}}\right)=\log\;\lambda_{0}+\beta_{\mathrm{eff}}\left[\log\left(\text{effor}t_{\mathrm{jk}}\right)\right]+\beta_{\mathrm{sex}}\left(\mathrm{se}x_{i}\right)-f\left[\text{dist}\left(i,j\right)|\theta ,\sigma_{\mathrm{sex}}\right]$$

where $f\left[\text{dist}\left(i,j\right)|\theta ,\sigma_{\mathrm{sex}}\right]$ describes how detection rate is a function of distance between the activity centre of individual i and pixel j, which are conditional on θ and $\sigma_{\mathrm{sex}}$.

**TABLE 1:** Five candidate models used to estimate the lion population abundance in Pilanesberg National Park, using a Bayesian SCR approach (Elliot et al., 2020).

Model 1—*N*(.), *λ*_0_(sex + effort), σ(sex): The basal encounter rate and the spatial scale parameter is sex‐specific Model 2—*N*(.), *λ*_0_(effort), σ(sex): The spatial scale parameter is sex‐specific, but the basal encounter rate is independent of sex Model 3—*N*(.), *λ*_0_(effort), σ(.): The spatial scale parameter and the basal encounter rate are independent of sex Model 4—*N*(.), *λ*_0_(sex + effort), σ(.): The spatial scale parameter is independent of sex, but the basal encounter rate is sex‐specific Model 5—N(.), *λ*_0_(effort): This is a conventional nonspatial capture–recapture model, corrected for effort

We ran the models using R (R Core Team, 2021) and the code provided by Elliot and Gopalaswamy (2017), which implements a Bayesian Markov Chain Monte Carlo (MCMC) procedure using the Metropolis–Hastings algorithm (Tierney, 1994). We ran 31,000 iterations per chain and set four chains for each model with an initial burn in of 1000 iterations. We assessed convergence using the Gelman–Rubin diagnostic and assumed convergence if the r‐hat value was <1.05 for each parameter (Gelman & Rubin, 1992). If nonconvergence persisted, we discarded more initial iterations, or we reran the analysis with more iterations. To select a model to report, we used two criteria. First, a goodness‐of‐fit evaluation, using the Bayesian *p*‐value based on individual encounters (Royle et al., 2009), was used to reject models whose *p*‐value lay outside the extremities (between .15 and .85). Second, we visually assessed pair‐wise correlation plots of the posterior outputs to assess parameter redundancy. All R scripts, functions, and data for our analysis are available in Appendix S1.

### 2.5 Assessing precision and bias associated with reduced effort

We repeated the analyses described above using subsamples of the empirical data to better understand how reduced sampling would affect the bias and precision of our estimates. To do this, we depleted our full empirical data set of 7068 km by ~1000 km increments, providing us with six subsets in addition to the complete data set. For each subset, we only retained the detections associated with the incremental drive effort, and to mimic a realistic sampling situation, we retained complete tracks, which typically require starting and ending at a base. Each increment of 1000 km was roughly equivalent to driving all road segments twice, and each subset of data has roughly uniform coverage. For these subsets, we only ran Model 1, which assumes that both the basal encounter rate $\left(\lambda_{0}\right)$ and the rate of decline in detection probability $\left(\sigma \right)$ are sex‐specific. We chose this model since it provides estimates on population size, sex ratio and sex‐specific movement, parameters, which are used for management decisions. To assess precision and relative bias of these estimates, we compared estimates from the reduced data sets to those based on the full empirical data set. Precision was measured by calculating the coefficient of variation using $\mathrm{CV}=\hat{\mathrm{SE}}(\hat{D})/\hat{D}$, and relative bias was calculated using $\mathrm{RB}=\left(\hat{D}-D\right)/D$, where D is the density estimate (assumed to be the true density) from the full empirical data set and $\hat{D}$ is the density estimate from a reduced data set. We considered data sets that produced estimates for σ, $\psi_{\mathrm{sex}}$ and $N_{\text{super}}$ with a CV < 20% and relative bias <15% to have good precision and minimal bias.

## 3 RESULTS

We sampled on 90 days during the 108‐day survey period and drove 7068 km in search of lions. We recorded 260 lion detections but discarded 61 detections for which we did not have adequate photographs to unambiguously identify the individuals and discarded a further 15 detections of lions deemed to be under 1 year of age. This gave us a total of 184 detections. From these detections, we identified 37 unique lions comprising 17 females (89 detections) and 20 males (95 detections), while the average number of traps within which each individual was detected (average spatial recaptures) was 4.6.

### 3.1 Model diagnostics

All models achieved convergence ($\hat{R}\leq 1.05$ for each parameter) with 30,000 iterations; although for Model 2 (1600) and Models 3 and 4 (1400), we did discard additional iterations post hoc. Bayesian *p*‐values were between .67 and .7, indicating all were adequate. The pair‐wise correlation plots showed minimal parameter redundancy across the models (Figure S1). These evidences, in addition to parameter estimates being very similar across all SCR models (Table S2), led us to report the estimates from Model 1. Detailed summaries of all models are provided in Tables S1 and S2.

### 3.2 Lion abundance, density, home range size

**FIGURE 3:**
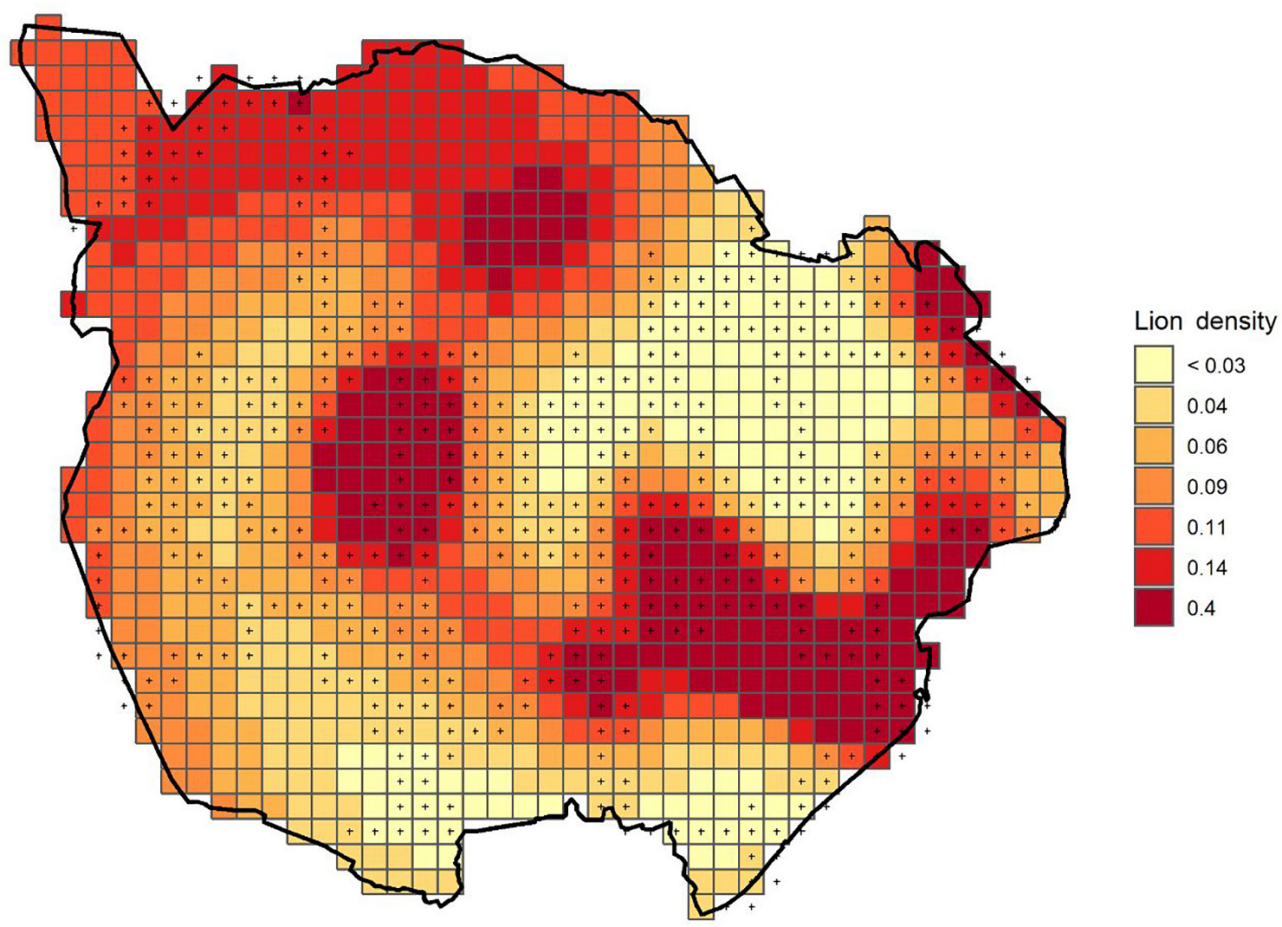
Pixel‐specific lion density estimated by Model 1 and expressed in units of individual lion activity centres per state‐space pixel (0.5 km^2^) in Pilanesberg National Park, South Africa. + Symbol denotes traps that were sampled.

Based on Model 1 (Table 2), the PNP lion population size was estimated to be 44 individuals over the age of 1 year (posterior SD = 3.05, 95% highest posterior density [HPD] interval = 38–49). Posterior mean lion density was 8.8/100 km^2^ (posterior SD = 0.6%, 95% HPD interval = 7.8–10.1). The estimated sex ratio produced by $\psi_{\mathrm{sex}}$ was 0.9*♀*:1♂. The estimate of σ for males (3.85, PSD = 0.33) and females (3.19, PSD = 0.27) translates to a home range estimate of 282 km^2^ (PSD = 49.1) for males and 193 km^2^ (PSD = 33.6) for females. Posterior density estimates for each 0.5 km^2^ pixel illustrate the primary ‘hotspots’ of lion activity (Figure 3).

**TABLE 2:** Posterior summaries of parameters estimated using a Bayesian spatial capture–recapture model to estimate spatial lion density in Pilanesberg National Park, South Africa.

Parameters	Posterior mean	Posterior standard deviation	95% lower HPD	95% upper HPD
*σ*_F_ – Rate of decline in detection rate (DR) as a female lion's activity centre increases as a function of her distance from the centroid of the sample grid cell	3.19	0.27	2.68	3.73
*σ*_M_ – Rate of decrease in DR as a male lion's activity centre increases as a function of his distance from the centroid of the sample grid cell	3.85	0.33	3.23	4.52
*β*_sex_ – Difference of complementary log–log value of DR between ♂ and *♀*	−0.13	0.25	−0.61	0.35
*β*_eff_ – Rate of change in the complementary log–log value of DR as the (log) effort changes by one unit	0.79	0.11	0.57	1.00
*λ*_0_ – Basal encounter rate of an individual (female for sex‐specific models) lion whose activity centre is located precisely at the centroid of the grid cell	0.005	0.001	0.003	0.007
*ψ* – Ratio of true number of individuals in the population compared with the data augmented population *M*	0.22	0.03	0.16	0.29
*ψ*_sex_ – Proportion of lions are male	0.53	0.08	0.37	0.69
*N*_super_ – Overall number of lions in larger state space	43.85	3.05	38	49
*D* – Estimated density of adult lion/100 km^2^	8.81	0.61	7.84	10.05

*Note*: Estimates presented below are from Model 1 $\left(\beta_{\mathrm{sex}},\theta \left(.\right)\left[.\right]\right)$ and include posterior standard deviations and 95% highest posterior density intervals (HPD). Number of posterior samples used was 30,000. Maximum value of potential scale reduction factor = 1, Bayesian *p*‐value = .7. See Figure S1 for pairwise plots of parameters, and Tables S1 and S2 for more detailed summaries from all models.

### 3.3 Assessing precision and bias associated with reduced effort

**FIGURE 4:**
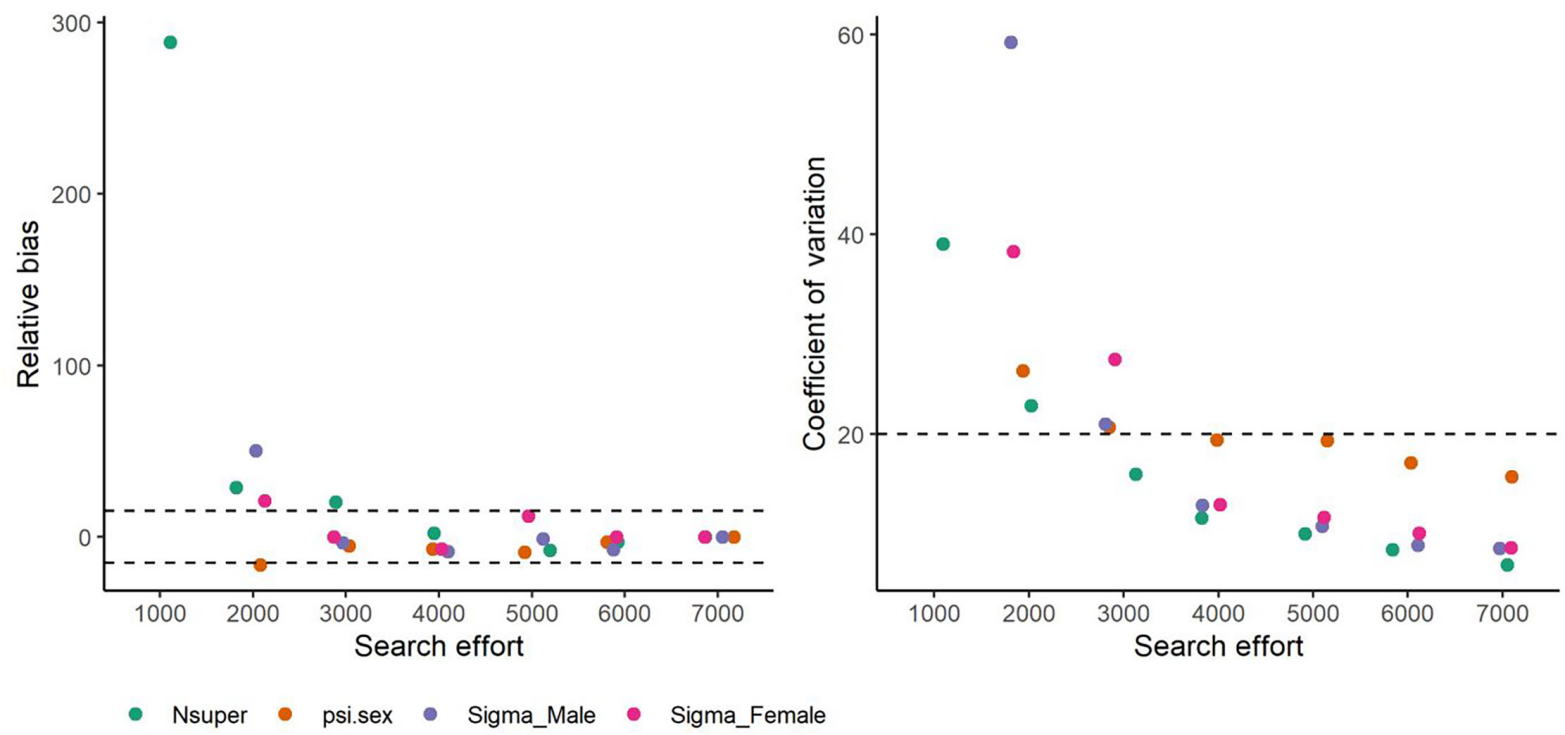
Total data set consisted of 7000 km of search effort to collect individual encounter histories on lions. To assess how bias (relative bias) and precision (measured by the coefficient of variation) were influenced by reduced sampling intensity, the data were sampled in 1000 km increments and analysed. Estimates of four key parameters are displayed (see Table 2 for definitions) and the dashed lines represent ±15% for relative bias, and 20% for coefficient of variation.

As 1000 km was incrementally removed from our empirical data set, the capture histories were diminished. The poorest data set consisted of the first 1000 km search effort and resulted in 18 individuals, with only two recaptures, both of which were female. As such, for this data set, we only ran Model 3 since it has no sex specificity. After 2000 km, the capture history had increased substantially with 28 individuals and 29 recaptures. For both data sets, we were forced to increase the number of iterations to 100,000 and retain only three chains (1000 km subset) and two chains (2000 km subset) to achieve an $\hat{R}\leq 1.1$. The 3000 km data set consisted of 30 individuals with 46 recaptures. All three of these subsets (1000, 2000, 3000 km) resulted in posterior estimates with low precision and high levels of relative bias for the key parameters of interest (σ, $\psi_{\mathrm{sex}}$ and $N_{\text{super}}$; Figure 4, Table S1). At 4000 km of search effort, which resulted in 31 individuals with 81 recaptures and 3.3 average spatial recaptures, precision was relatively high (CV < 20%) and relative bias was low (RB < 15%) for all parameters of interest. Both additional subsets (5000 and 6000 km) together with the full empirical data set, had increasing precision and decreasing relative bias (Figure 4, Table S3). Posterior density for each 0.5 km^2^ pixel varied considerably, particularly among the three poorest data sets, but was relatively stable between 4000 and 7000 km (Figure S2).

## 4 DISCUSSION

Our estimate of 8.8 individuals per 100 km^2^ is somewhat lower than the figure of 11.36 provided by Packer, Loveridge, et al. (2013), and our abundance estimate (44 lions) was also lower than the 2001 figure of 50 lions provided by Tambling and Du Toit (2005), who estimated an annual growth rate of 10.6% between 1994 and 2001. Note that lion numbers in fenced parks in South Africa is often highly manipulated by management authorities (see Ferreira & Hofmeyr, 2014), which is also the case in PNP. These figures, combined with field perceptions based on prey decline, had led the park management to anticipate a much higher estimate, as they had expressed concern over an abnormally high lion density in the park. However, our estimates suggest that lion density is similar to other fenced wildlife areas (Elliot et al., 2020, 2021), and considerably lower than some free ranging populations (Elliot & Gopalaswamy, 2017).

In a review of 40 scientific papers, Périquet et al. (2014) reported that on average lions had a sex ratio of 2.3*♀*:1♂, while also recognising that many of these studies have not applied contemporary, robust methods to estimate sex ratio. We estimated the sex ratio in PNP to be close to parity (0.9♀:1♂). This is consistent with emerging evidence in fenced lion populations (Elliot et al., 2020, 2021). The equal sex ratio in PNP may be a consequence of the inability of males to disperse far from their natal home ranges which could result in males staying within the confines of the park and being relieved of dispersal‐related mortality events (Elliot, Valeix, Macdonald, & Loveridge, 2014). However, it could also be the result of previous management interventions (e.g. removal of lions) and requires further study. Regardless, the effects of an equal sex ratio on prey consumption need to be considered since PNP management expressed concerns about the need to control the lion population to limit further prey species declines. Considering that male lions eat more than females (Fritz et al., 2011), an obvious management recommendation would be to attempt to maintain a 2*♀*:1♂ sex ratio. Prey depletion in fenced parks such as PNP is, however, a complex issue potentially caused by multiple factors.

Our finding that males move more, and have larger home ranges than females, with different detection rates, is consistent with other studies and validates the use of sex‐specific models where possible (Elliot, Cushman, Macdonald, & Loveridge, 2014; Elliot & Gopalaswamy, 2017). However, home range size (282 km^2^ for males and 193 km^2^), is quite large relative to the park size, suggesting a degree of overlap between social groups that is also seemingly apparent in the individual capture history (Figure 4). Furthermore, such large home ranges may also indicate relatively low prey densities within the park, which corroborated with our observations in the field at the time.

The results of our subsampling exercise suggest that for future surveys, field teams should aim to complete a minimum of 4000 km search effort to obtain accurate and precise estimates. This is almost half the field effort of our study, suggesting that the costs could be markedly reduced. However, we note that additional effort beyond 4000 km did increase the precision of the estimates, highlighting the trade‐off between expenditure of limited resources and obtaining reliable estimates. While our goal was to provide guidelines for future surveys in terms of expenditure (kilometres driven), it is the capture history itself that influences the bias and precision of results. After 4000 km of drive effort, we had recorded 31 individuals, 81 recaptures and 3.3 average spatial recaptures. During future surveys, field teams should look to these as minimum data objectives. We also demonstrate the importance of excessive sampling to account for both bias and precision when arriving at suggestions of optimal sample sizes. For example, recently, a faecal DNA‐based SE‐SECR approach was developed using only precision as a measure to draw inference on forest elephant (*Loxodonta* cyclotis) density (Laguardia, Gobush, et al., 2021), which was then applied nationally using a sampling design to estimate elephant abundance across Gabon (Laguardia, Bourgeois, et al., 2021). Results from our subsampling exercise indicate that this estimate may be biased high.

In areas such as PNP (fenced and small, with habituated lions), the SECR approach is highly applicable to monitoring lions, and we urge its application to other small fenced protected areas where reliable estimates are not available yet are required for intensive management. We note that the current study provides yet another example where lion abundance was thought to be considerably higher than rigorous estimates suggest (Elliot et al., 2020, 2021), and we urge wildlife managers to undertake systematic surveys using cutting‐edge methods prior to making important decisions relating to lions. Importantly in our study, despite intensive sampling, our analysis suggests that we detected ~84% of the available individuals, which is still not a ‘whole count’. We suggest that this monitoring should occur on an annual basis so that in time estimates of vital rates and population trend can be obtained.

## AUTHOR CONTRIBUTIONS

**Isabella A. Ball:** Conceptualization (equal); data curation (equal); formal analysis (lead); investigation (lead); methodology (lead); project administration (supporting); writing – original draft (lead); writing – review and editing (supporting). **David G. Mareweck:** Conceptualization (supporting); data curation (equal); formal analysis (supporting); methodology (supporting); supervision (supporting); writing – original draft (equal); writing – review and editing (equal). **Nicholas B. Elliot:** Conceptualization (equal); formal analysis (equal); investigation (supporting); methodology (equal); supervision (supporting); writing – original draft (supporting); writing – review and editing (equal). **Arjun M. Gopalaswamy:** Conceptualization (equal); formal analysis (equal); investigation (supporting); methodology (equal); supervision (supporting); validation (equal); writing – original draft (supporting); writing – review and editing (equal). **Herve Fritz:** Conceptualization (equal); formal analysis (supporting); investigation (supporting); supervision (supporting); writing – original draft (supporting); writing – review and editing (supporting). **Jan A. Venter:** Conceptualization (equal); funding acquisition (lead); investigation (supporting); methodology (supporting); project administration (lead); resources (lead); supervision (lead); writing – original draft (equal); writing – review and editing (equal).

## Supporting information


Appendix S1.

## Data Availability

The data are available at: Venter (2023), ‘African lions (*Panthera leo*) SECR – 2020’, Mendeley Data, V1, doi: 10.17632/472ypb4ckn.1.

## References

[ece310291-bib-0001] Bauer, H. , Dickman, A. , Chapron, G. , Oriol‐Cotterill, A. , Nicholson, S. K. , Sillero‐Zubiri, C. , Hunter, L. , Lindsey, P. , & Macdonald, D. W. (2020). Threat analysis for more effective lion conservation. Oryx, 56(1), 1–8. 10.1017/S0030605320000253

[ece310291-bib-0002] Braczkowski, A. , Gopalaswamy, A. M. , Elliot, N. B. , Possingham, H. P. , Bezzina, A. , Maron, M. , Biggs, D. , & Allan, J. R. (2020). Restoring Africa's lions: Start with good counts. Frontiers in Ecology and Evolution, 8, 1–3, 10.3389/fevo.2020.00138

[ece310291-bib-0003] Braczkowski, A. , Gopalaswamy, A. M. , Nsubuga, M. , Allan, J. , Biggs, D. , & Maron, M. (2020). Detecting early warnings of pressure on an African lion (*Panthera leo*) population in the Queen Elizabeth Conservation Area, Uganda. Ecological Solutions and Evidence, 1, e12015. 10.1002/2688-8319.12015

[ece310291-bib-0004] Carruthers, J. (2011). Pilanesberg National Park, North West Province, South Africa: Uniting economic development with ecological design – a history, 1960s to 1984. Koedoe: African Protected Area Conservation and Science, 53, 1–10.

[ece310291-bib-0005] Clark, T. , Horne, J. S. , Hebblewhite, M. , & Luis, A. D. (2021). Stochastic predation exposes prey to predator pits and local extinction. Oikos, 130, 300–309.

[ece310291-bib-0006] Creel, S. , Becker, M. S. , Durant, S. M. , M'Soka, J. , Matandiko, W. , Dickman, A. J. , Christianson, D. , Dröge, E. , Mweetwa, T. , Pettorelli, N. , Rosenblatt, E. , Schuette, P. , Woodroffe, R. , Bashir, S. , Beudels‐Jamar, R. C. , Blake, S. , Borner, M. , Breitenmoser, C. , Broekhuis, F. , … Zimmermann, A. (2013). Conserving large populations of lions – The argument for fences has holes. Ecology Letters, 16, 1413, e1–3. 10.1111/ele.12145 23837659

[ece310291-bib-0007] Elliot, N. B. , Bett, A. , Chege, M. , Sankan, K. , de Souza, N. , Kariuki, L. , Broekhuis, F. , Omondi, P. , Ngene, S. , & Gopalaswamy, A. M. (2020). The importance of reliable monitoring methods for the management of small, isolated populations. Conservation Science and Practice, 2, e217. 10.1111/csp2.217

[ece310291-bib-0008] Elliot, N. B. , Broekhuis, F. , Omondi, P. , Ngene, S. , Kariuki, L. , Sankan, K. , Chege, M. , Wato, Y. , Amoke, I. , Dolrenry, S. , & Gopalaswamy, A. M. (2021). Report on the application of novel estimating methodologies to monitor lion abundance within source populations and large carnivore occupancy at a national scale . Wildlife Research and Training Institute and Kenya Wildlife Service. ISBN: 978‐9914‐40‐516‐3.

[ece310291-bib-0009] Elliot, N. B. , Cushman, S. A. , Loveridge, A. J. , Mtare, G. , & Macdonald, D. W. (2014). Movements vary according to dispersal stage, group size, and rainfall: The case of the African lion. Ecology, 95, 2860–2869. 10.1890/13-1793.1

[ece310291-bib-0010] Elliot, N. B. , Cushman, S. A. , Macdonald, D. W. , & Loveridge, A. J. (2014). The devil is in the dispersers: Predictions of landscape connectivity change with demography. Journal of Applied Ecology, 51, 1169–1178. 10.1111/1365-2664.12282

[ece310291-bib-0011] Elliot, N. B. , & Gopalaswamy, A. M. (2017). Towards accurate and precise estimates of lion density. Conservation Biology, 31, 934–943. 10.1111/cobi.12878 27958641

[ece310291-bib-0012] Elliot, N. B. , Valeix, M. , Macdonald, D. W. , & Loveridge, A. J. (2014). Social relationships affect dispersal timing revealing a delayed infanticide in African lions. Oikos, 123, 1049–1056. 10.1111/oik.01266

[ece310291-bib-0013] Ferreira, S. M. , & Funston, P. J. (2010). Estimating lion population variables: Prey and disease effects in Kruger National Park, South Africa. Wildlife Research, 37(3), 194–206.

[ece310291-bib-0014] Ferreira, S. M. , & Hofmeyr, M. (2014). Managing charismatic carnivores in small areas: Large felids in South Africa. South African Journal of Wildlife Research, 44(1), 32–42.

[ece310291-bib-0015] Fritz, H. , Loreau, M. , Chamaillé‐Jammes, S. , Valeix, M. , & Clobert, J. (2011). A food web perspective on large herbivore community limitation. Ecography, 34, 196–202.

[ece310291-bib-0016] Gelman, A. , & Rubin, D. B. (1992). Inference from iterative simulation using multiple sequences. Statistical Science, 7, 457–472.

[ece310291-bib-0017] Gopalaswamy, A. M. , Delampady, M. , Karanth, K. U. , Kumar, N. S. , & Macdonald, D. W. (2015). An examination of index‐calibration experiments: Counting tigers at macroecological scales. Methods in Ecology and Evolution, 6, 1055–1066. 10.1111/2041-210x.12351

[ece310291-bib-0018] Gopalaswamy, A. M. , Elliot, N. B. , Ngene, S. , Broekhuis, F. , Braczkowski, A. , Lindsey, P. , Packer, C. , & Stenseth, N. C. (2022). How “science” can facilitate the politicization of charismatic megafauna counts. Proceedings of the National Academy of Sciences, 119, e2203244119. 10.1073/pnas.2203244119 PMC9171922 35544693

[ece310291-bib-0019] Karanth, K. U. , & Nichols, J. D. (1998). Estimating tiger densities in India using photographic captures and recaptures. Ecology, 79, 2852–2862.

[ece310291-bib-0020] Kissui, B. M. , & Packer, C. (2004). Top–down population regulation of a top predator: Lions in the Ngorongoro crater. Proceedings of the Royal Society of London Series B: Biological Sciences, 271, 1867–1874. 10.1098/rspb.2004.2797 PMC1691790 15315904

[ece310291-bib-0021] Laguardia, A. , Bourgeois, S. , Strindberg, S. , Gobush, K. S. , Abitsi, G. , Ateme, H. B. B. , Ebouta, F. , Fay, J. M. , Gopalaswamy, A. M. , Maisels, F. , & Daouda, E. S. B. (2021). Nationwide abundance and distribution of African forest elephants across Gabon using non‐invasive SNP genotyping. Global Ecology and Conservation, 32, e01894.

[ece310291-bib-0022] Laguardia, A. , Gobush, K. S. , Bourgeois, S. , Strindberg, S. , Abitsi, G. , Ebouta, F. , Fay, J. M. , Gopalaswamy, A. M. , Maisels, F. , Ogden, R. , & White, L. J. (2021). Assessing the feasibility of density estimation methodologies for African forest elephant at large spatial scales. Global Ecology and Conservation, 27, e01550.

[ece310291-bib-0023] Le Roux, E. , Marneweck, D. G. , Clinning, G. , Druce, D. J. , Kerley, G. I. , & Cromsigt, J. P. (2019). Top–down limits on prey populations may be more severe in larger prey species, despite having fewer predators. Ecography, 42, 1115–1123.

[ece310291-bib-0024] McEvoy, O. K. (2019). *The management of lions (*Panthera leo*) in small, fenced wildlife reserves* . [Doctoral dissertation, PhD thesis]. Rhodes University, Grahamstown, South Africa.

[ece310291-bib-0025] Miller, S. , Bissett, C. , Burger, A. , Courtenay, B. , Dickerson, T. , Druce, D. , Ferreira, S. , Funston, P. , Hofmeyr, D. , & Kilian, P. (2013). Management of reintroduced lions in small, fenced reserves in South Africa: An assessment and guidelines. South African Journal of Wildlife Research, 43, 138–154.

[ece310291-bib-0026] Miller, S. M. , & Funston, P. J. (2014). Rapid growth rates of lion (*Panthera leo*) populations in small, fenced reserves in South Africa: A management dilemma. African Journal of Wildlife Research, 44, 43–55.

[ece310291-bib-0027] Miller, S. M. , & Funston, P. J. (2016). Aging the African lion: A training on aging lions, version 1 . http://agingtheafricanlion.org

[ece310291-bib-0028] Mucina, L. , & Rutherford, M. C. (2006). The vegetation of South Africa, Lesotho and Swaziland. South African National Biodiversity Institute.

[ece310291-bib-0029] Ngene, S. , Broekhuis, F. , Elliot, N. B. , Mukeka, J. , Chege, M. , Muteti, D. , Ngoru, B. , Lala, F. , Mwiu, S. , & Amoke, I. (2023). The emergence of a robust and inclusive framework for a nationwide assessment of a African lions. Conservation Science and Practice, 5(2), e12871.

[ece310291-bib-0030] Otis, D. L. , Burnham, K. P. , White, G. C. , & Anderson, D. R. (1978). Statistical inference from the capture data on closed animal populations. Wildlife Monographs, 62, 1–135.

[ece310291-bib-0031] Packer, C. , Loveridge, A. , Canney, S. , Caro, T. , Garnett, S. T. , Pfeifer, M. , Zander, K. K. , Swanson, A. , MacNulty, D. , Balme, G. , Bauer, H. , Begg, C. M. , Begg, K. S. , Bhalla, S. , Bissett, C. , Bodasing, T. , Brink, H. , Burger, A. , Burton, A. C. , … Polasky, S. (2013). Conserving large carnivores: Dollars and fence. Ecology Letters, 16, 635–641. 10.1111/ele.12091 23461543

[ece310291-bib-0032] Packer, C. , Swanson, A. , Canney, S. , Loveridge, A. , Garnett, S. , Pfeifer, M. , Burton, A. C. , Bauer, H. , & MacNulty, D. (2013). The case for fencing remains intact. Ecology Letters, 16, 1414‐e4. 10.1111/ele.12171 23962143

[ece310291-bib-0033] Pekor, A. , Miller, J. R. B. , Flyman, M. V. , Kasiki, S. , Kesch, M. K. , Miller, S. M. , Uiseb, K. , van der Merve, V. , & Lindsey, P. A. (2019). Fencing Africa's protected areas: Costs, benefits, and management issues. Biological Conservation, 229, 67–75. 10.1016/j.biocon.2018.10.030

[ece310291-bib-0034] Pennycuick, C. J. , & Rudnai, J. (1970). A method of identifying individual lions (*Panthera leo*), with an analysis of reliability of identification. Journal of Zoology, 160, 497–508.

[ece310291-bib-0035] Périquet, S. , Fritz, H. , & Revilla, E. (2014). The lion king and the hyaena queen: Large carnivore interactions and coexistence. Biological Reviews, 90, 1197–1214. 10.1111/brv.12152 25530248

[ece310291-bib-0036] R Core Team . (2021). R: A language and environment for statistical computing. R Foundation for Statistical Computing.

[ece310291-bib-0037] Royle, J. A. , Chandler, R. B. , Sollmann, R. , & Gardner, B. (2013). Spatial capture–recapture. Academic Press. 10.1016/C2012-0-01222-7

[ece310291-bib-0038] Royle, J. A. , Karanth, K. U. , Gopalaswamy, A. M. , & Kumar, N. S. (2009). Bayesian inference in camera trapping studies for a class of spatial capture–recapture models. Ecology, 90, 3233–3244. 19967878 10.1890/08-1481.1

[ece310291-bib-0039] Stoffelen, A. , Adiyia, B. , Vanneste, D. , & Kotze, N. (2020). Post‐apartheid local sustainable development through tourism: An analysis of policy perceptions among ‘responsible’ tourism stakeholders around Pilanesberg National Park, South Africa. Journal of Sustainable Tourism, 28, 414–432.

[ece310291-bib-0040] Tambling, C. J. , & Du Toit, J. T. (2005). Modelling wildebeest population dynamics: Implications of predation and harvesting in a closed system. Journal of Applied Ecology, 42, 431–441.

[ece310291-bib-0041] Tierney, L. (1994). Markov chains for exploring posterior distributions. The Annals of Statistics, 22(4), 1701–1728.

[ece310291-bib-0042] Van Dyk, G. , & Slotow, R. (2003). The effects of fences and lions on the ecology of African wild dogs reintroduced to Pilanesberg National Park, South Africa. African Zoology, 38, 79–94.

[ece310291-bib-0043] Vanak, A. T. , Thaker, M. , & Slotow, R. (2010). Do fences create an edge‐effect on the movement patterns of a highly mobile mega‐herbivore? Biological Conservation, 143, 2631–2637. 10.1016/j.biocon.2010.07.005

[ece310291-bib-1043] Venter, J. (2023). African lions (Panthera leo) SECR – 2020, Mendeley Data, V1. 10.17632/472ypb4ckn.1

[ece310291-bib-0044] Western, G. , Elliot, N. B. , Sompeta, S. L. , Broekhuis, F. , Ngene, S. , & Gopalaswamy, A. M. (2022). Lions in a coexistence landscape: Repurposing a traditional field technique to monitor an elusive carnivore. Ecology and Evolution, 12, e8662. 10.1002/ece3.8662 35261749 PMC8888262

