# Supplementary material for: Considerations on effort, precision and accuracy for long‐term monitoring of African lions (Panthera leo), when using Bayesian spatial explicit capture–recapture models, in fenced protected areas
Source: Ecol Evol. 2023 Jul 17;13(7):e10291. doi: 10.1002/ece3.10291 (PMC10352093; doi:10.1002/ece3.10291)

Figure S1. Pairwise plots between estimated parameters from the posterior MCMC draws. We used these to visually assess covariance and parameter redundancy (or identifiability) issues as a result of model overfitting relative to sample size. Since the ecological parameters, abundance (*N_super_*) and sex ratio ($\psi_{sex})$, were of primary interest in our study, we were particularly concerned to assess whether there were correlations between these parameters and any other parameter.

Model 1: $N\left( . \right),\lambda_{0}\left( sex+effort \right), \sigma(sex)$


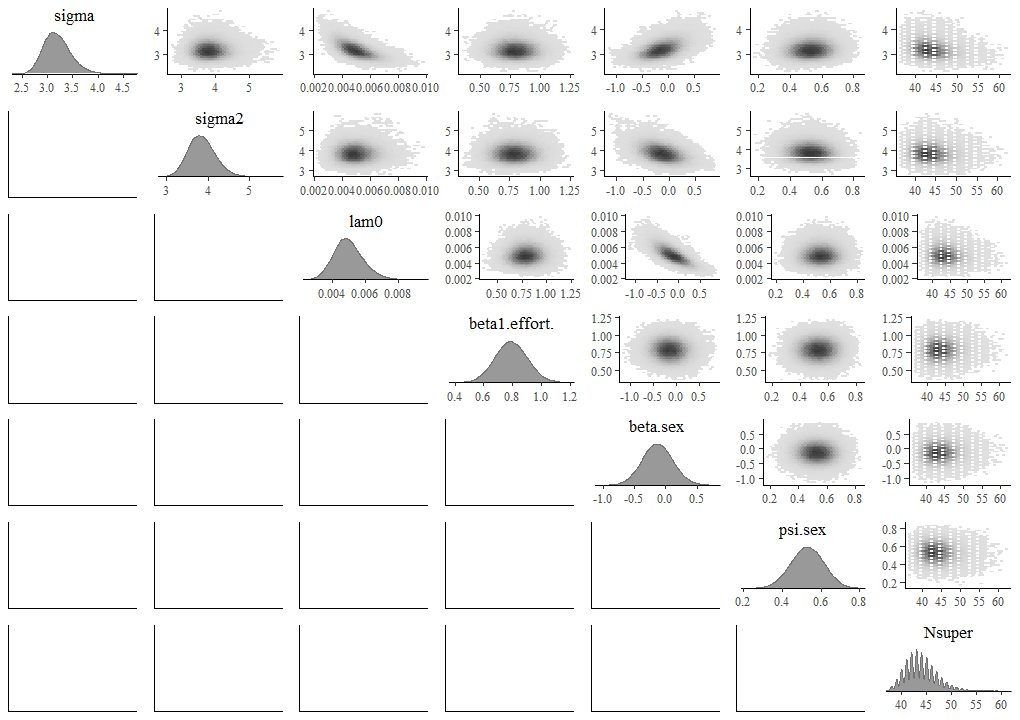


Model 2: $N\left( . \right),\lambda_{0}\left( effort \right), \sigma(sex)$


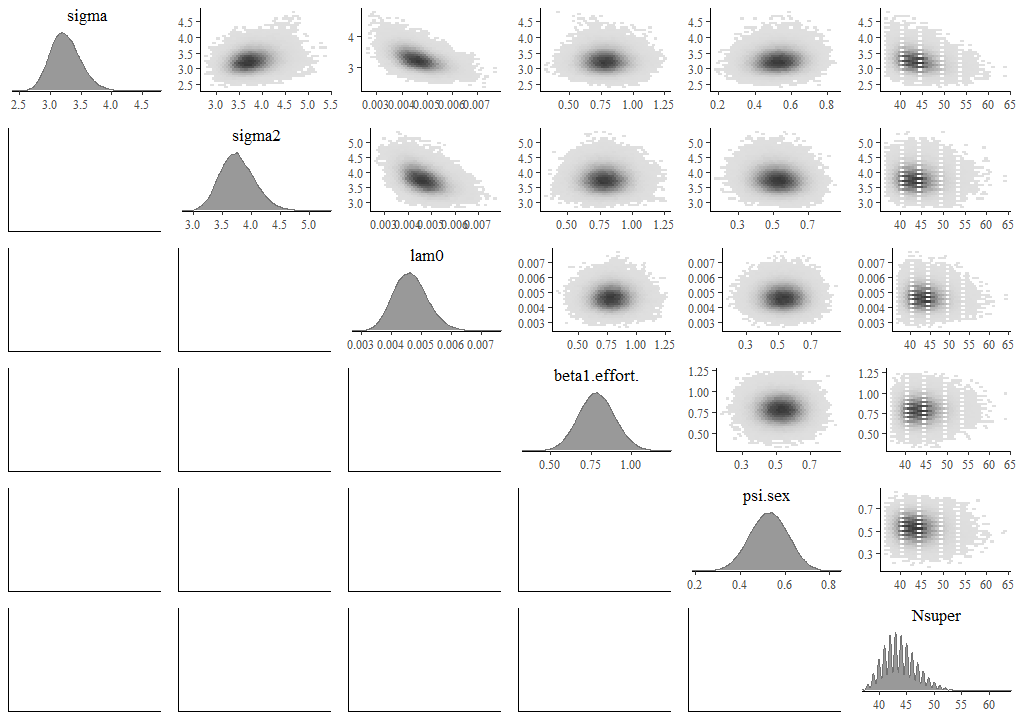


Model 3: $N\left( . \right),\lambda_{0}\left( effort \right), \sigma\left( . \right)$


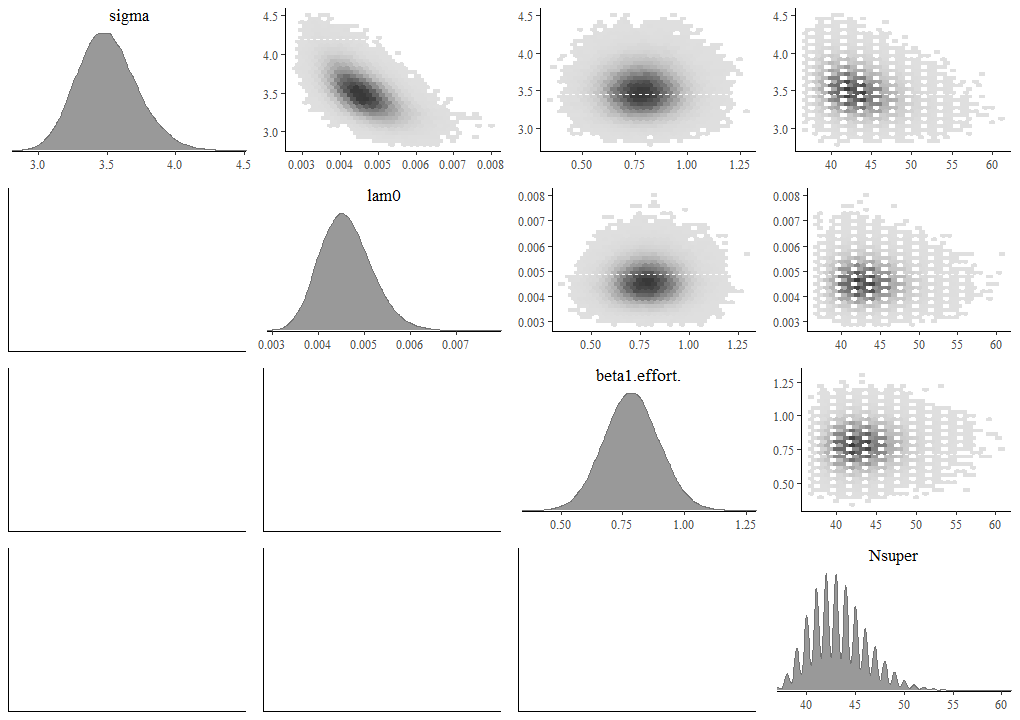


Model 4: $N\left( . \right),\lambda_{0}\left( sex+effort \right), \sigma(.)$


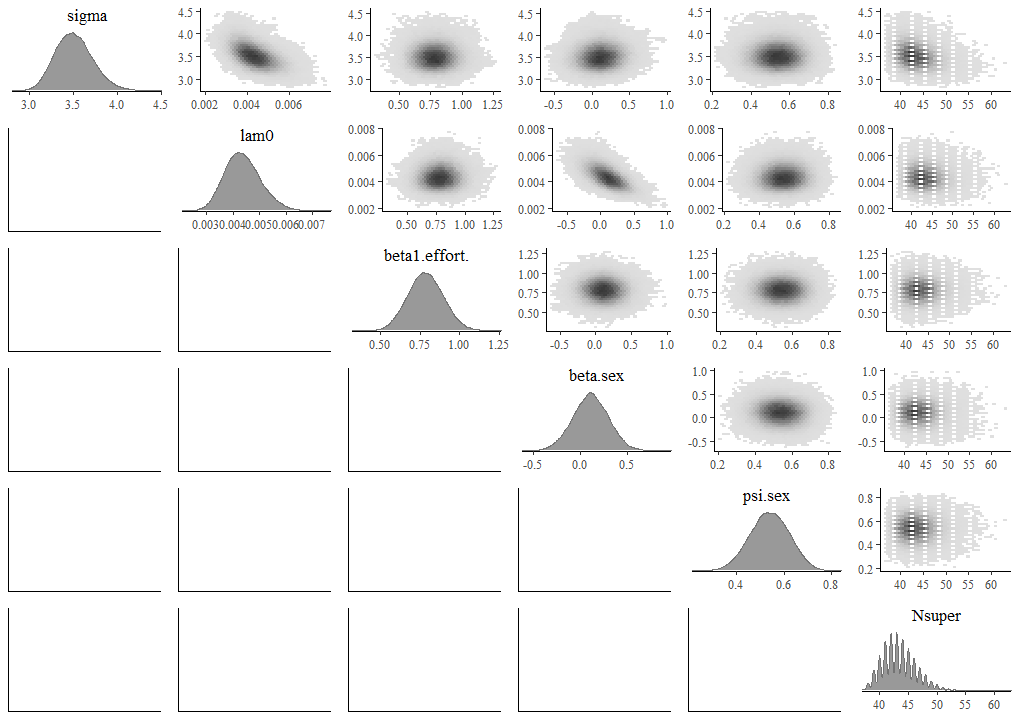


Table S.1. Model specifications and diagnostics for models 1-5.

|  | Model 1 | Model 2 | Model 3 | Model 4 | Model 5 |
| --- | --- | --- | --- | --- | --- |
| Model Specifications | **Setting** | **Setting** | **Setting** | **Setting** | **Setting** |
| M_sex_ | 1 | 0 | 0 | 1 | 0 |
| M_sexsigma_ | 1 | 1 | 0 | 0 | 0 |
| M_sigma_ | 1 | 1 | 1 | 1 | 0 |
| Theta | 1 | 1 | 1 | 1 | 1 |
| Nz | 163 | 163 | 163 | 163 | 163 |
| Chains | 4 | 4 | 4 | 4 | 4 |
| Iterations | 31,000 | 31,000 | 31,000 | 31,000 | 31,000 |
| Burn | 1,000 | 1,000 | 1,000 | 1,000 | 1,000 |
| Post Hoc Burn | 0 | 1,600 | 1,400 | 1,400 | 0 |
| Model Diagnostics |  |  |  |  |  |
| Bayes P-Value | 0.696 | 0.686 | 0.672 | 0.679 |  |

Table S.2. Posterior estimates of parameters for Models 1-5. Model 1 was selected for reporting due to (a) Bayesian *p* value lying within the extremities (0.15-0.85), (b) minimal pairwise correlations.

Model 1: $N\left( . \right),\lambda_{0}\left( sex+effort \right), \sigma(sex)$

|  | Mean | Monte Carlo SE | Median | Mode | Post. SD | Lower 95% HPDI | Upper 95% HPDI |
| --- | --- | --- | --- | --- | --- | --- | --- |
| $\sigma_{F}$  $\sigma_{M}$ | 3.19 | 0.01 | 3.17 | 3.08 | 0.27 | 2.68 | 3.73 |
|  | 3.85 | 0.01 | 3.83 | 3.76 | 0.33 | 3.23 | 4.52 |
| $\lambda_{0}$ | 0.005 | 0.005 | 0.005 | 0.001 | 0.000 | 0.003 | 0.007 |
| $\beta_{eff}$ | 0.79 | 0.00 | 0.79 | 1.11 | 0.11 | 0.57 | 1.00 |
| $\beta_{sex}$ | -0.13 | 0.01 | -0.13 | -0.33 | 0.25 | -0.61 | 0.35 |
| $\psi$ | 0.22 | 0.00 | 0.22 | 0.19 | 0.03 | 0.16 | 0.29 |
| $\psi_{sex}$ | 0.53 | 0.00 | 0.53 | 0.59 | 0.08 | 0.37 | 0.69 |
| $N_{super}$ | 43.85 | 0.03 | 44.00 | 43.00 | 3.05 | 38.00 | 49.00 |
| $D$ | 8.81 | 0.01 | 8.84 | 8.64 | 0.61 | 7.84 | 10.05 |

Model 2: $N\left( . \right),\lambda_{0}\left( effort \right), \sigma\left( sex \right)$

|  | Mean | Monte Carlo SE | Median | Mode | Post. SD | Lower 95% HPDI | Upper 95% HPDI |
| --- | --- | --- | --- | --- | --- | --- | --- |
| $\sigma_{F}$  $\sigma_{M}$ | 3.25 | 0.00 | 3.23 | 3.59 | 0.25 | 2.79 | 3.74 |
|  | 3.77 | 0.01 | 3.75 | 4.19 | 0.30 | 3.19 | 4.37 |
| $\lambda_{0}$ | 0.005 | 0.004 | 0.005 | 0.001 | 0.000 | 0.004 | 0.006 |
| $\beta_{eff}$ | 0.79 | 0.00 | 0.78 | 0.70 | 0.11 | 0.57 | 1.01 |
| $\beta_{sex}$ | NA | NA | NA | NA | NA | NA | NA |
| $\psi$ | 0.22 | 0.00 | 0.22 | 0.26 | 0.03 | 0.16 | 0.29 |
| $\psi_{sex}$ | 0.53 | 0.00 | 0.53 | 0.48 | 0.08 | 0.37 | 0.69 |
| $N_{super}$ | 43.86 | 0.03 | 44.00 | 43.00 | 3.08 | 38.00 | 49.00 |
| $D$ | 8.82 | 0.01 | 8.84 | 8.64 | 0.62 | 7.84 | 10.05 |

Model 3: $N\left( . \right),\lambda_{0}\left( effort \right), \sigma\left( . \right)$

|  | Mean | Monte Carlo SE | Median | Mode | Post. SD | Lower 95% HPDI | Upper 95% HPDI |
| --- | --- | --- | --- | --- | --- | --- | --- |
| $\sigma_{F}$  $\sigma_{M}$ | 3.50 | 0.01 | 3.49 | 3.23 | 0.22 | 3.09 | 3.94 |
|  |  |  |  |  |  |  |  |
| $\lambda_{0}$ | 0.005 | 0.000 | 0.005 | 0.005 | 0.001 | 0.004 | 0.006 |
| $\beta_{eff}$ | 0.79 | 0.00 | 0.78 | 0.77 | 0.11 | 0.57 | 1.00 |
| $\beta_{sex}$ | NA | NA | NA | NA | NA | NA | NA |
| $\psi$ | 0.22 | 0.00 | 0.22 | 0.18 | 0.03 | 0.16 | 0.28 |
| $\psi_{sex}$ | NA | NA | NA | NA | NA | NA | NA |
| $N_{super}$ | 43.32 | 0.04 | 43.00 | 42.00 | 2.90 | 37.00 | 48.00 |
| $D$ | 8.71 | 0.01 | 8.64 | 8.44 | 0.58 | 7.84 | 10.05 |

Model 4: $N\left( . \right),\lambda_{0}\left( sex+effort \right), \sigma\left( . \right)$

|  | Mean | Monte Carlo SE | Median | Mode | Post. SD | Lower 95% HPDI | Upper 95% HPDI |
| --- | --- | --- | --- | --- | --- | --- | --- |
| $\sigma_{F}$  $\sigma_{M}$ | 3.51 | 3.57 | 3.50 | 0.21 | 0.01 | 3.10 | 3.94 |
|  |  |  |  |  |  |  |  |
| $\lambda_{0}$ | 0.004 | 0.000 | 0.004 | 0.005 | 0.001 | 0.003 | 0.006 |
| $\beta_{eff}$ | 0.78 | 0.00 | 0.78 | 0.49 | 0.11 | 0.56 | 1.00 |
| $\beta_{sex}$ | 0.11 | 0.00 | 0.11 | -0.07 | 0.19 | -0.27 | 0.48 |
| $\psi$ | 0.22 | 0.00 | 0.22 | 0.23 | 0.03 | 0.16 | 0.29 |
| $\psi_{sex}$ | 0.54 | 0.00 | 0.54 | 0.53 | 0.08 | 0.37 | 0.70 |
| $N_{super}$ | 43.45 | 0.04 | 43.00 | 42.00 | 2.97 | 38.00 | 49.00 |
| $D$ | 8.73 | 0.01 | 8.64 | 8.44 | 0.60 | 7.84 | 10.05 |

Model 5: $N\left( . \right),\lambda_{0}\left( effort \right)$

|  | Mean | Monte Carlo SE | Median | Mode | Post. SD | Lower 95% HPDI | Upper 95% HPDI |
| --- | --- | --- | --- | --- | --- | --- | --- |
| $\sigma_{F}$  $\sigma_{M}$ | NA | NA | NA | NA | NA | NA | NA |
|  |  |  |  |  |  |  |  |
| $\lambda_{0}$ | 0.001 | 0.000 | 0.001 | 0.001 | 0.000 | 0.001 | 0.001 |
| $\beta_{eff}$ | 0.77 | 0.00 | 0.77 | 0.97 | 0.11 | 0.56 | 0.98 |
| $\beta_{sex}$ | NA | NA | NA | NA | NA | NA | NA |
| $\psi$ | 0.19 | 0.00 | 0.19 | 0.18 | 0.03 | 0.14 | 0.25 |
| $\psi_{sex}$ | NA | NA | NA | NA | NA | NA | NA |
| $N_{super}$ | 37.30 | 0.00 | 37.00 | 37.00 | 0.56 | 37.00 | 38.00 |
| $D$ | 7.50 | 0.00 | 7.44 | 7.44 | 0.11 | 7.44 | 7.64 |

|  | Search Effort Invested (km) | | | | | | | | | | | | | | | | | | | | | | |  |  |  |
| --- | --- | --- | --- | --- | --- | --- | --- | --- | --- | --- | --- | --- | --- | --- | --- | --- | --- | --- | --- | --- | --- | --- | --- | --- | --- | --- |
|  | | 1000 | | | 2000 | | | 3000 | | | 4000 | | | 5000 | | | | 6000 | | | | | 7000 | | | |
| Dataset summaries | |  | | |  | | |  | | |  | | |  | | | |  | | | | |  | | | |
| Number of individuals | | 18 | | | 28 | | | 30 | | | 31 | | | 31 | | | | 34 | | | | | 37 | | | |
| Number of recaptures | | 2 | | | 29 | | | 46 | | | 81 | | | 103 | | | | 123 | | | | | 147 | | | |
| Average spatial recaptures | | 1.11 | | | 2.04 | | | 2.37 | | | 3.29 | | | 4.00 | | | | 4.26 | | | | | 4.59 | | | |
| Model specification | |  | | |  | | |  | | |  | | |  | | | |  | | | | |  | | | |
| Msex | | 0 | | | 1 | | | 1 | | | 1 | | | 1 | | | | 1 | | | | | 1 | | | |
| Msexsigma | | 0 | | | 1 | | | 1 | | | 1 | | | 1 | | | | 1 | | | | | 1 | | | |
| M | | 300 | | | 200 | | | 200 | | | 200 | | | 200 | | | | 200 | | | | | 200 | | | |
| Iterations | | 100k | | | 100k | | | 30k | | | 30k | | | 30k | | | | 30k | | | | | 30k | | | |
| Chains | | 5 | | | 5 | | | 4 | | | 4 | | | 4 | | | | 4 | | | | | 4 | | | |
| Model diagnostics | |  | | |  | | |  | | |  | | |  | | | |  | | | | |  | | | |
| Post-hoc burn | | 0 | | | 40k | | | 0 | | | 0 | | | 0 | | | | 0 | | | | | 0 | | | |
| Chains retained | | 3 | | | 2 | | | 4 | | | 4 | | | 4 | | | | 4 | | | | | 4 | | | |
| Max Gelman Ruben | | 1.08 | | | 1.1 | | | 1 | | | 1 | | | 1 | | | | 1 | | | | | 1 | | | |
| Bayes p-value | | 0.5 | | | 0.5 | | | 0.5 | | | 0.7 | | | 0.7 | | | | 0.8 | | | | | 0.7 | | | |
| Posterior parameter estimates (presented as mean, relative bias, coefficient of variation) | | | | | | | | | | | | | | | | | | | | | | | | | | |
| $\sigma_{F}$ | | 5.3 | 37 | 89 | 4.8 | 21 | 38 | 3.1 | 0 | 28 | 2.9 | -7 | 13 | 3.2 | 12 | 12 | 3.0 | | 0 | | 10 | | 3.2 | | . | 9 |
| $\sigma_{M}$ | | 5.3 | 65 | 89 | 4.7 | 50 | 59 | 3.9 | -3 | 21 | 3.6 | -9 | 13 | 4.3 | -1 | 11 | 3.9 | | -7 | | 9 | | 3.9 | | . | 9 |
| $\beta_{sex}$ | | . | . | . | 0.1 | . | . | -0.4 | . | . | -0.1 | . | . | -0.2 | . | . | -0.3 | | . | | . | | -0.1 | | . | . |
| $\beta_{eff}$ | | 0.2 | . | . | 0.6 | . | . | 0.9 | . | . | 0.6 | . | . | 0.7 | . | . | 0.8 | | . | | . | | 0.8 | | . | . |
| $\lambda_{0}$ | | 0.001 | . | . | 0.003 | . | . | 0.004 | . | . | 0.006 | . | . | 0.005 | . | . | 0.006 | | | . | | . | 0.005 | | . | . |
| $\psi$ | | 0.6 | . | . | 0.3 | . | . | 0.3 | . | . | 0.2 | . | . | 0.2 | . | . | 0.2 | | . | | . | | 0.2 | | . | . |
| $\psi_{sex}$ | | . | . | . | 0.4 | -16 | 26 | 0.5 | -5 | 21 | 0.5 | -7 | 19 | 0.5 | -9 | 19 | 0.5 | | -3 | | 17 | | 0.5 | | . | 16 |
| $N_{super}$ | | 170.3 | 288 | 39 | 56.6 | 29 | 23 | 52.8 | 20 | 16 | 44.8 | 2 | 12 | 40.5 | -8 | 10 | 42.6 | | -3 | | 8 | | 43.9 | | . | 7 |

Table S3. To provide guidelines on resource allocation (km search effort) required for robust estimates of population parameters for lions in Pilanesberg National Park, we rarefied our full empirical dataset (7,000 km) by 1,000 km increments, and analysed the reduced datasets using Bayesian spatial capture-recapture models. This table presents the data summaries, model specification, model diagnostics and posterior summary estimates for each incremental subset of the data. See manuscript for definitions of parameters.

Figure S2.  Pixel-specific lion density expressed in units of individual lion activity centres per state-space pixel (0.5 km^2^) in Pilanesberg National Park. We rarefied our full empirical dataset (7,000 km) by 1,000 km increments and analysed the reduced datasets using Bayesian spatial capture-recapture models. This figure illustrates how inferences on spatial distribution will vary markedly if inadequate datasets are used.


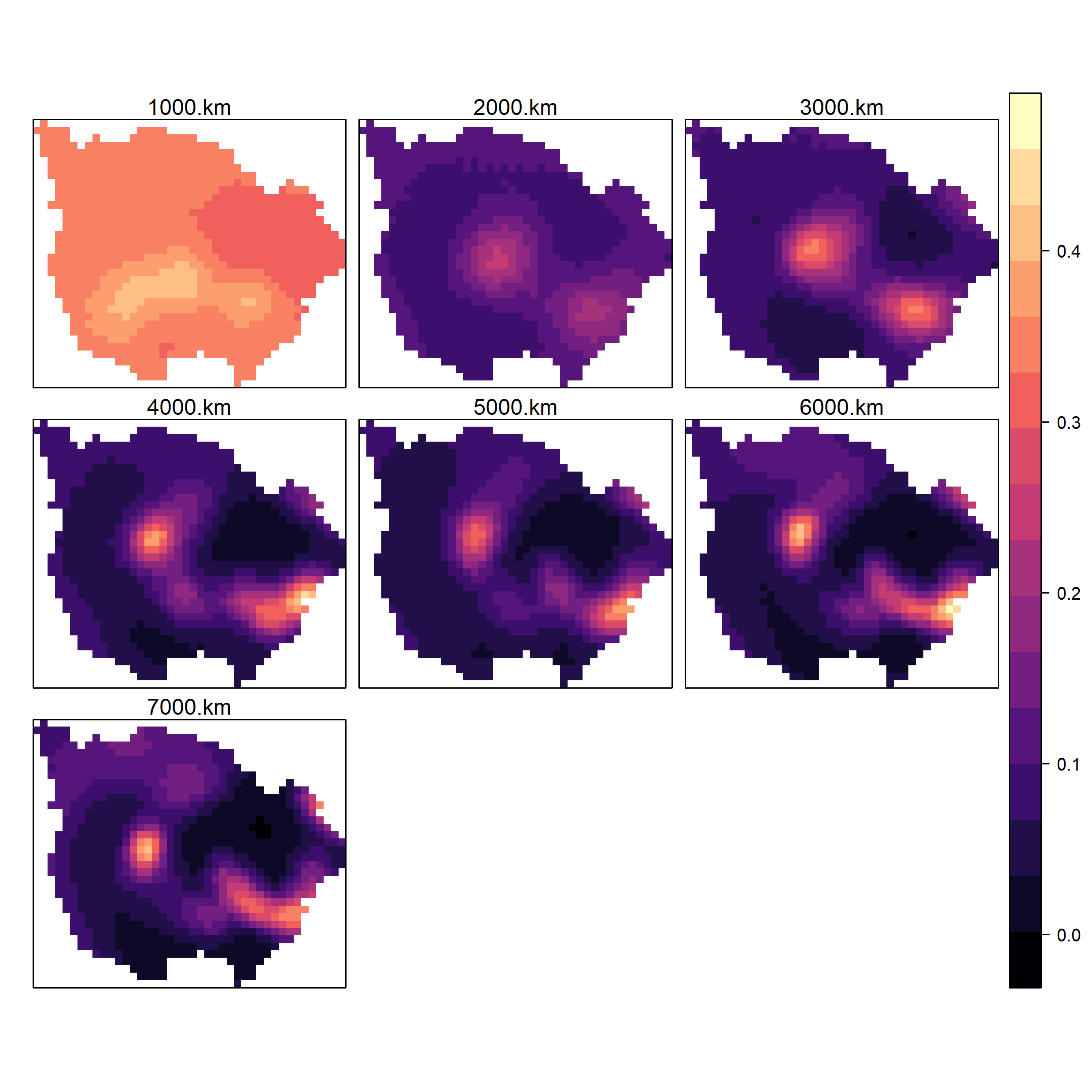

Supplement: Supplementary file 1 — Appendix S1. [file ECE3-13-e10291-s001.docx]
